# Autosomal dominant Kenny-Caffey syndrome with congenital hypoparathyroidism, short stature and normal intellect: a case report

**DOI:** 10.1186/1687-9856-2015-S1-P72

**Published:** 2015-04-28

**Authors:** Mary Abraham, Li Dong, Shoshana Rath, Susan O’Connell, Fiona McKenzie, Hakon Hakonarson, Catherine S Choong, Michael Levine

**Affiliations:** 1 Department of Endocrinology, Princess Margaret Hospital, Perth, WA, Australia; 2 Center for Applied Genomics, Abramson Research Center, The Children’s Hospital of Philadelphia, USA; 3 School of Paediatrics and Child Health, UWA, Perth, WA, Australia; 4 Genetic Services of Western Australia, Princess Margaret Hospital & King Edward Memorial Hospital, Perth, WA, Australia; 5 Division of Human Genetics and Department of Pediatrics, The Children’s Hospital of Philadelphia and The Perelman School of Medicine, USA; 6 Division of Pulmonary Medicine, The Children’s Hospital of Philadelphia, USA; 7 Division of Endocrinology and Diabetes, The Children’s Hospital of Philadelphia, USA; 8 Center for Bone Health, The Children’s Hospital of Philadelphia, USA

## 

Kenny-Caffey syndrome (KCS) is characterized by proportionate short stature, cortical thickening and medullary stenosis of tubular bones, delayed closure of anterior fontanelle, eye abnormalities, and hypoparathyroidism. The autosomal dominant form (KCS Type 2) caused by mutations in FAM111A is distinguished from the autosomal recessive form (KCS Type1), caused by mutations in TBCE gene, by the absence of mental retardation.

Our proband presented on day 8 of life with hypocalcaemic seizures secondary to hypoparathyroidism. Normocalcaemia was achieved with IV calcium gluconate and maintained by oral calcium carbonate 100mg BD and calcitriol 0.1mcg BD for the first 2 years of life while serum PTH remained low at <0.3pmol/L. There has been no evidence of nephrocalcinosis on follow up. The dose of supplemental calcium and calcitriol is being gradually reduced. She also has persistent mild microcytic anaemia with normal iron stores.

Her phenotype included small hands and feet, triangular hypoplastic and dystrophic nails, hypoplastic mid-face, macrocrania and large persistent fontanelles. Karotype and FISH for 22q11 deletion were normal. Initial investigations included a normal ECHO, renal ultrasound and MRI brain.

Her neurodevelopment is normal but her growth is compromised. At 1 year, her length was 65.5cms (SDS -2.9) and at 3 years, her height was 80cms (SDS -3.8). There is no family history of short stature or hypoparathyroidism. She is growth hormone sufficient on pharmacological testing; however she has been commenced on growth hormone treatment based on her poor growth velocity and short stature. It is currently too early to determine response.

A skeletal survey performed at 2 years of age was suggestive of KCS. Genetic testing revealed a heterozygous mutation c.1622C>A (p.Ser541Tyr) in FAM111A. However, the unusual nails, the reducing calcium requirement and the unexplained microcytic anaemia are unique to our patient.

Written informed Consent for this patient has been taken including results of the genetic analyses and images according to the Institutional Ethics Committee procedures of our health service.

